# The Cytotoxic Activity and Metabolic Profiling of *Hyptis rhomboidea* Mart. et Gal

**DOI:** 10.3390/molecules29174216

**Published:** 2024-09-05

**Authors:** Jian Zhang, Wenjie Gao, Israt Jahan, Run Zhai, Kaiwei Yao, Jian Yan, Ping Li

**Affiliations:** Key Laboratory of Agro-Environment in the Tropics, College of Natural Resources and Environment, South China Agricultural University, Guangzhou 510642, China; zj1607106714@163.com (J.Z.); gaowenjie0902@163.com (W.G.); isratanu35@gmail.com (I.J.); 13184571757@163.com (R.Z.); 18657017035@163.com (K.Y.)

**Keywords:** *Hyptis rhomboidea*, human cancer cells, cytotoxicity, metabolite profiling, UPLC-QTOF-MS, GC-MS

## Abstract

Many naturally occurring chemical metabolites with significant cytotoxic activities have been isolated from medicinal plants and have become the leading hotspot of anti-cancer research in recent years. *Hyptis rhomboidea* Mart. et Gal is used as a folk medicine in South China to treat or assist in the treatment of liver disease, ulcers, and edema. But its chemical constituents have not been fully investigated yet. This study aimed to assess the cytotoxicity of *H. rhomboidea*, which was chemically characterized by chromatography–mass spectrometry methods. The results showed that the 95% ethanol extract of *H. rhomboidea* has marked inhibitory effects on five human cancer cell lines (HL-60, A549, SMMC-7721, MDA-MB-231, and SW480), with IC_50_ values ranging from 15.8 to 40.0 μg/mL. A total of 64 compounds were identified by ultra-high-performance liquid chromatography with quadrupole time-of-flight mass spectrometry (UPLC-QTOF-MS) and gas chromatograph–mass spectroscopy (GC-MS) analysis of *H. rhomboidea* crude extract. Among them, kaempferol, quercetin, rosmarinic acid, squalene, and campesterol were found to be abundant and might be the major metabolites involved to its bioactivity. The cytotoxic characterization and metabolite profiling of *H. rhomboidea* displayed in this research provides scientific evidence to support its use as medicinal properties.

## 1. Introduction

Cancer is one of the most dangerous diseases, posing a significant threat to human health worldwide. According to GLOBOCAN 2020 statistics on cancer incidence and mortality, there were approximately 19.3 million new cancer cases and nearly 10 million cancer deaths in 2020 [1]. Among them, breast cancer (2.3 million), lung cancer (2.24 million), colorectal cancer (1.97 million), prostate cancer (1.44 million), and stomach cancer (1.1 million) are still high-incidence diseases, with lung cancer causing the highest number of deaths annually. Despite substantial advancements in cancer biology, oncology, and surgical techniques in recent years, the overall survival rate for cancer has only slightly improved over the past decades, and this disease remains one of the leading causes of death [2,3,4]

Currently, the main treatment methods for cancer include radiotherapy, chemotherapy, and surgery [5]. While these treatments can be effective, the toxicity of anti-cancer drugs and the development of drug resistance in cancer cells often diminish their efficacy and result in significant side effects, such as nausea and loss of appetite [6,7,8]. Research has discovered that some natural plant compounds possess significant cytotoxicity against cancer cells lines, and they are abundantly available and have few toxicity side effects [9]. Therefore, natural plant compounds are important raw ingredients for exploring novel anti-cancer drugs. Combining natural anti-cancer compounds with conventional chemotherapy drugs can help to jointly inhibit cancer cell growth, prevent the development of drug resistance in tumor cells, and reduce the adverse side effects associated with chemotherapy [10]. A total of 185 small anticancer drugs were approved by the U.S. Food and Drug Administration (FDA) for their anticancer activity from 1946 to 2019. Among them, 75 small-molecule drugs (41% of the total) originated from natural products and their derivatives [11]. Therefore, natural products play significant role in new drug discovery and development.

**Figure 1:**
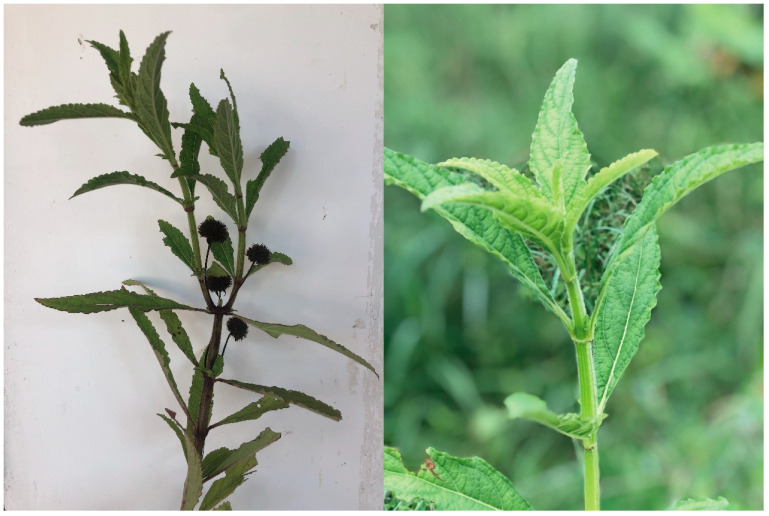
Exterior view of *Hyptis rhomboidea* Mart. et Gal.

*Hyptis rhomboidea* Mart. et Gal (Figure 1), also called *H*. *decurrens*, *Pycnanthemum decurrens*, is a medicinal plant belonging to the Lamiaceae family, native to tropical and subtropical regions of North America and widely distributed in the coastal areas of South China [12,13]. It is used as Chinese folk medicine to assist in the treatment of hepatitis, ulcers, and edema [14]. Phytochemical and pharmacological studies revealed that *H. rhomboidea* mainly contains phenylpropanoids, terpenoids, and phenolic acid. For example, hyprhombin C and epi-hyprhombin B (first identified in *H. rhomboidea*) demonstrate excellent anti-xanthine oxidase activity [15]. Caryophyllene oxide can be used to relieve pain without inducing psychoactive side effects, while ursolic acid is effective in treating autoimmune arthritis [16,17]. However, there are no reports about the cytotoxic activity of *H. rhomboidea* in human cancer cells.

In this study, the cytotoxic activities of *H. rhomboidea* ethanol extract against five human cancer cell lines including leukemia (HL-60), hepatoma (SMMC-7721), lung cancer (A-549), breast cancer (MDA-MB-231), and colon cancer (SW480) were assessed by the MTS method [18]. Based on the activity screening results, the metabolic profiling of *H. rhomboidea* extract was further analyzed using ultra-high-performance liquid chromatography coupled to quadrupole time-of-flight mass spectrometry (UPLC-QTOF-MS) and gas chromatography–mass spectrometry (GC-MS). This analysis helped to explore the potential cytotoxicity of natural products from the medicinal plant *H. rhomboidea* against human cancer cell lines.

## 2. Results

### 2.1. Cytotoxic Activity Evaluation of H. rhomboidea Crude Extracts

To investigate the cytotoxic activity of ethanol extracts from *H. rhomboidea*, five types of human cancer cells (HL-60, A549, SMMC-7721, MDA-MB-231, and SW480) were incubated with 100 µg/mL of the extracts. The cell viability of the cultured cancer cells was measured using MTS assays, evaluated the cytotoxicity of 95% and 50% ethanol extracts of *H. rhomboidea* against the five human cancer cell lines.

**Figure 2:**
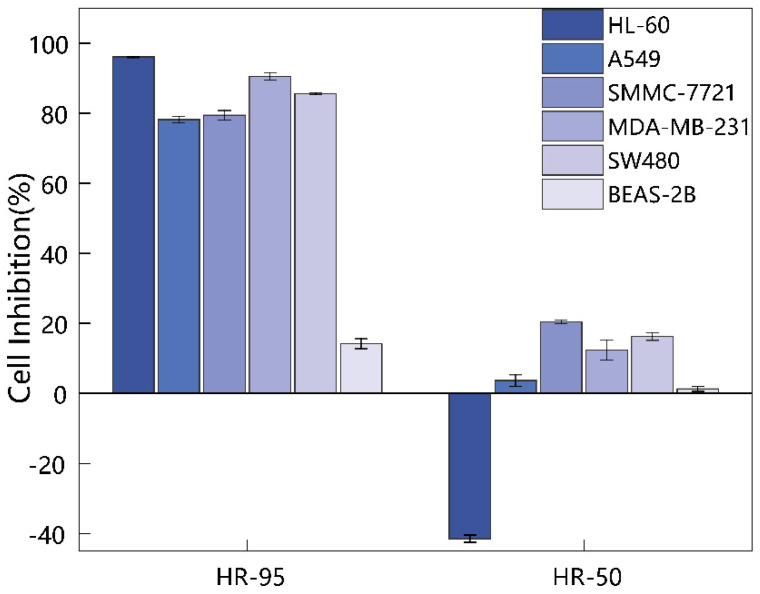
Cytotoxic activity of the 95% ethanol extract (HR-95) and the 50% ethanol extract (HR-50) of *H. rhomboidea* on five human cancer cell lines. The five human cancer cell lines include leukemia cells (HL-60), hepatoma cells (A549), lung cancer cells (SMMC-7721), breast cancer cells (MDA-MB-231), colon cancer cells (SW480), and human normal lung epithelial cells (BEAS-2B). The extract concentration was 100 μg/mL, and the value means the cell inhibition rate ± SD of cancer cells (*n* = 3).

The 95% ethanol extract (HR-95) exhibited significant cytotoxic activity against the growth of the five types of cancer cells in vitro. Compared with normal human cells (BEAS-2B), HR-95 exhibited a significantly higher inhibitory effect against the five human tumor cell types (Figure 2). Notably, the extract was particularly effective in inhibiting the growth of leukemia (HL-60) and breast cancer (MDA-MB-231) cells, with rates of 96.10% and 90.54%, respectively. The inhibition rates against hepatoma (A549), lung cancer (SMMC-7721), and colon cancer (SW480) cell growth were 78.24%, 79.43%, and 85.64%, respectively. In contrast, the 50% ethanol extract (HR-50) had low inhibitory effect on cell viability and promoted HL-60 cell growth by 41.52%. For the negative control, both extracts exhibited no cytotoxic activity against the growth of human normal lung epithelial cells (BEAS-2B), with inhibition rates of only 14.20% and 1.57%, respectively (Table 1).

### 2.2. Screening of the 95% Ethanol Extract for Cytotoxic Activity against Human Cancer Cell Lines

After assessing the cytotoxic activity of *H. rhomboidea* (HR-95), it was observed that HR-95 exhibits obviously inhibitory activity, with half-maximal effectiveness against the growth of the five human cancer cell lines in vitro. Therefore, the cell viability of cancer cells was determined using the MTS method at different concentrations of HR-95 (0, 0.8, 4, 20, 100 μg/mL). Positive control compounds included different concentrations of cisplatin and Taxol.

**Figure 3:**
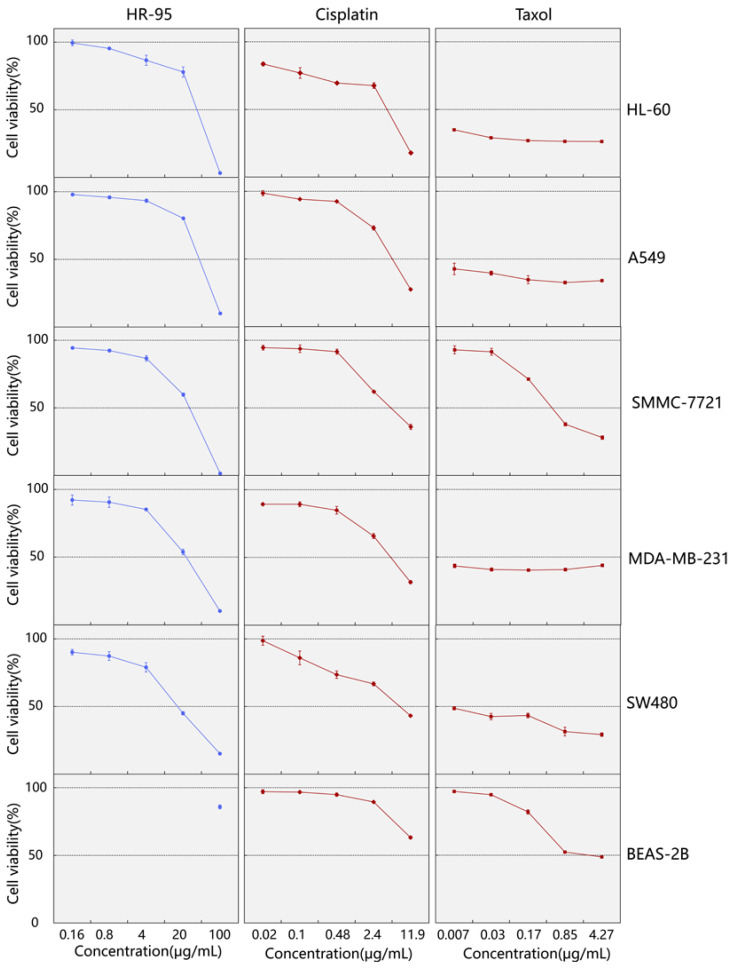
The cell viability curves of five human cancer cell lines (HL-60, A549, SMMC-7721, MDA-MB-231, and SW480) and normal human lung epithelial cells (BEAS-2B) treated with different concentrations of HR-95, cisplatin, and Taxol. HR-95: 95% ethanol extract of *H. rhomboidea*. The concentration of HR-95 for BEAS-2B was only 100 μg/mL. The x-axis represents the compound concentrations, with a maximum concentration of 100 μg/mL for HR-95, 12.00 μg/mL (40 μM) for cisplatin, and 4.27 μg/mL (5 μM) for Taxol. The y-axis represents the mean ± standard deviation of cell viability (*n* = 3).

After 48 h of incubation, cell viability assays were performed to evaluate the cytotoxic effects of HR-95 on the five human cancer cell lines. HR-95 and the positive control cisplatin exhibited poor inhibitory effects on the proliferation of human normal lung epithelial cells (BEAS-2B), and HR-95 possessed satisfactory cytotoxic activity against the five human cancer cell lines compared with the positive control cisplatin, with particularly noteworthy inhibitory effects on breast cancer (MDA-MB-231) and colon cancer cells (SW480) (Figure 3). Based on IC_50_ calculations, HR-95 exhibited distinct half-maximal inhibitory concentrations against the following five cancer cell lines: HL-60 (36.44 ± 1.82 μg/mL), A549 (40.02 ± 0.58 μg/mL), SMMC-7721 (26.20 ± 0.77 μg/mL), MDA-MB-231 (22.95 ± 1.59 μg/mL), and SW480 (15.81 ± 0.63 μg/mL) (Table 2). The results indicate that HR-95 contains compounds with potential cytotoxic activity against human cancer cell lines and warrants further isolation and identification for characterization.

### 2.3. Metabolic Profiling of H. rhomboidea

The results of the MTS assay showed that the 95% ethanol extract of the whole *H. rhomboidea* (HR-95) plant had great cytotoxic activity, while 50% extract had no inhibitory activity. To elucidate the chemical constituents (HR-95) with cytotoxic activity, LC-MS and GC-MS analysis were performed to characterize the metabolite profiles.

#### 2.3.1. Non-Volatile Chemical Compound Analysis

**Figure 4:**
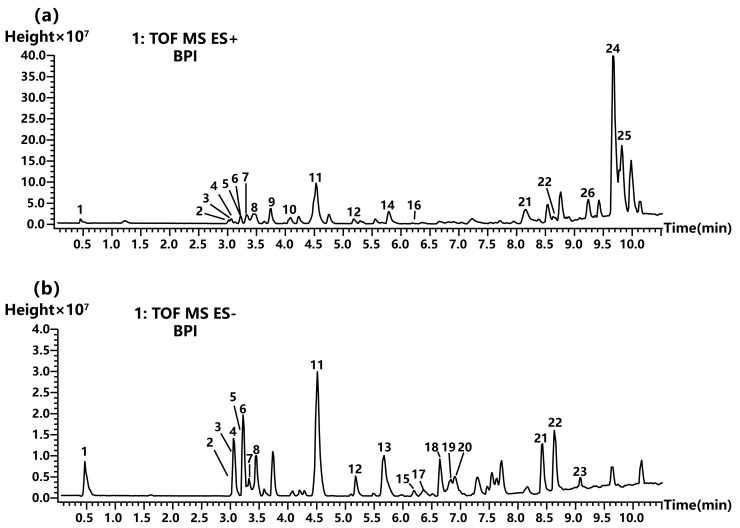
Base peak chromatograms of the 95% ethanol extract of *H. rhomboidea* (HR-95). (**a**) Base peak chromatograms of the positive ion mode. (**b**) Base peak chromatograms of the negative ion mode. Compound peaks are ranked by number.

To identify the non-volatile compositions of HR-95, UPLC-QTOF-MS conditions were optimized to establish the suitable elution gradient, ratio of formic acid in the mobile phase, operating voltage, and other parameters and achieve optimal chromatographic peak intension and separation. The identification of metabolites was performed by combining the positive and negative ion modes to obtain base peak chromatograms. The results were retained at the initial 10 min, and chromatographic peaks found in blank samples were excluded to enhance the accuracy of the analysis (Figure 4). Different types of metabolites were detected between positive and negative ion modes, with the total peak intensity notably higher in positive ion mode than in negative ion mode.

Based on the retention time, high-resolution mass spectra molecular ions and the fragment ions of peaks were obtained from the low-energy and ramp collision energy of MS^E^ data. According to the rules of ion fragmentation, compared with open-source mass spectral libraries and the literature on the *Hyptis* genus, 25 compounds with relatively high contents including eleven flavonoids, six terpenoids, two coumarins, three aliphatic derivatives, two aromatic derivatives, and one saccharide were tentatively identified from HR-95 (Table 3).

**Figure 5:**
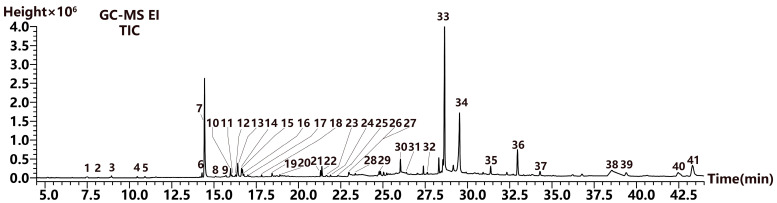
Total ion chromatogram of the 95% ethanol extract of *H. rhomboidea* (HR-95). Compound peaks were ranked by number.

Among the identified compounds, three types of flavonoids were categorized as follows: flavonols, flavones, and flavanes. These flavonoids exhibited strong molecular or quasi-molecular ion peaks using the ESI ion source. Peaks 4, 6, and 14 exhibited molecular ions [M + H]^+^ at *m*/*z* 303.0482, 287.0534, and 329.1004, respectively. The protonated molecular ion of the peak 4 *m*/*z* produced 285.0365 [M + H − 18], 257.0420 [M + H − 46], and 229.0467 [M + H − 74] ion fragments, identified as quercetin, and the inferred process for the cleavage is shown in Figure 5 [19]. Peak 6 generated ion fragments at *m*/*z* 213.0517 [M + H − 74], 153.0153 [M + H − 134], and 121.0255 [M + H − 166], identified as kaempferol [20]. While peak 14 produced ion fragments at *m*/*z* 314.0764 [M + H − 15], 296.0660 [M + H − 33], and 268.0708 [M + H − 61], identified as 3′,4′,5′-trimethoxyflavonol [21]. All of three compounds belong to flavonols.

Peaks 2 and 3 presented the molecular ions [M + H]^+^ at *m*/*z* 611.1622 and 465.1021, respectively. Nonetheless, they shared the same daughter ions of *m*/*z* 303.0482 (quercetin glycoside fragment), 229.0467, and 153.0151. Thus, peaks 2 and 3 were identified as rutin and spiraeoside [22,23]. Peaks 5 and 7 were detected in negative ion mode and exhibited the molecular ion [M − H]^−^ at *m*/*z* 593.1633 and 447.0931, respectively. Moreover, peak 5 produced fragment ions at *m*/*z* 285.0405 [M − H − 308], 255.0302 [M − H − 338], and 227.0351 [M − H − 366], which was identified as nicotiflorin [24]. Peak 7 possessed fragment ions at *m*/*z* 285.0325 [M − H − 163], 255.0296 [M − H − 192], and 227.0345 [M − H − 220], which was identified as astragalin [25]. These four compounds were classified as flavonol glycosides.

Peak 12 was observed the molecular ion [M − H]^−^ at *m*/*z* 283.0611, and the deprotonated molecule yielded several characteristic ion fragments at *m*/*z* 268.0373 [M − H − 15], 240.0415 [M − H − 43]; and 211.0393 [M − H − 72]; thus, it was speculated to be genkwanin [26]. Peak 16 was deduced as 5-Hydroxy-4′,7-dimethoxyflavone based on its molecular ion [M + H]^+^ at *m*/*z* 299.0893 and fragment ions at *m*/*z* 284.0655 [M + H − 15] and 256.0708 [M + H − 43] [27]. These two compounds were classified as flavones. Peak 9 and 11 belonged to flavanes, where peak 9 had a precursor ion [M + H]^+^ of *m*/*z* 423.1254 and a product ion [M + H − 60] of *m*/*z* 363.1036, compared with the pubchem database, and was tentatively identified as catechin 7-arabinofuranoside. Peak 11 produced a molecular ion [M − H]^−^ of *m*/*z* 453.0722 and fragment ions of *m*/*z* 329.2333 [M − H − 124] and 160.8415 [M − H − 293], which was inferred to be theaflavic acid based on the literature and databases [25].

Peaks 8 and 23 belonged to coumarins, and the molecular ions [M − H]^−^ were identified as rosmarinic acid and rutamarin at *m*/*z* 359.0769 and 355.1586, respectively. The possible identifications of the other 12 peaks are summarized in Table 3 and not described here.

**Table 3:** The chemical constituents in HR-95 tentatively identified by UPLC-QTOF-MS.

Peak No.	RT (min)	Molecular Weight (Da)	Putative Compound	Class	Molecular Formula	Fragment Ions *m*/*z*	Reference
1	0.488	378	Geshoidin	Saccharides	C_18_H_18_O_9_	377.0846/313.0864/173.8846/161.8413/160.8414	[28]
2	3.071	610	Rutin	Flavonoids	C_27_H_30_O_16_	519.0209/340.9951/303.0482/229.0467/153.0153	[22]
3	3.072	464	Spiraeoside	Flavonoids	C_21_H_20_O_12_	465.1013/303.0482/229.0467/153.0151/137.0202	[23]
4	3.141	302	Quercetin	Flavonoids	C_15_H_10_O_7_	303.0482/285.0369/257.0420/229.0467/153.0153	[19]
5	3.220	594	Nicotiflorin	Flavonoids	C_27_H_30_O_15_	593.1539/285.0405/255.0302/227.0351	[24]
6	3.221	286	Kaempferol	Flavonoids	C_15_H_10_O_6_	287.0539/213.0517/165.0154/153.0153/121.0255	[20]
7	3.289	448	Astragalin	Flavonoids	C_21_H_20_O_11_	447.0931/284.0325/255.0296/227.0345	[25]
8	3.454	360	Rosmarinic acid	Coumarins	C_18_H_16_O_8_	359.0773/197.0451/179.0346/161.0242/133.0290/134.0450	[29]
9	3.752	422	Catechin 7-arabinofuranoside	Flavonoids	C_20_H_22_O_10_	423.1254/363.1036	-
10	4.083	382	Resorcinolnaphthalein	Aromatic derivatives	C_24_H_14_O_5_	383.0852/347.1084/333.1290/287.0863/205.0828	-
11	4.547	428	Theaflavic acid	Flavonoids	C_21_H_16_O_10_	453.0722/329.2333/160.8415	[25]
12	5.193	284	Genkwanin	Flavonoids	C_16_H_12_O_5_	283.0607/268.0373/240.0415/211.0393/161.0239/151.0023	[26]
13	5.673	488	Uncaric acid	Terpenoids	C_30_H_48_O_5_	487.3456/469.3322/425.3420	-
14	5.793	312	3′,4′,5′-Trimethoxyflavonol	Flavonoids	C_18_H_16_O_6_	329.1001/314.0764/296.0660/268.0708	[21]
15	6.204	488	Asiatic acid	Terpenoids	C_30_H_48_O_5_	533.3480/487.3429	[30]
16	6.273	298	5-Hydroxy-4′,7-dimethoxyflavone	Flavonoids	C_17_H_14_O_5_	299.0893/284.0655/256.0708/184.0704	[31]
17	6.37	294	9-HOTrE	Aliphatic derivatives	C_18_H_30_O_3_	293.2122/275.9977/231.1745/171.9812	[32]
18	6.684	472	Maslinic acid	Terpenoids	C_30_H_48_O_4_	472.3516/471.3482/405.3159	[33]
19	6.788	296	α-Dimorphecolic acid	Aliphatic derivatives	C_18_H_32_O_3_	295.2278/277.2173/171.3940	[34]
20	6.902	472	Corosolic acid	Terpenoids	C_30_H_48_O_4_	472.3513/471.3484	[35]
21	8.159	456	Ursolic acid	Terpenoids	C_30_H_48_O_3_	455.3534/407.3311	[36]
22	8.628	334	4-Ethenyloctahydro-2-hydroxy-4,5′,8′a-trimethyl-1′-oxospiro[cyclopentane-1,2′(1′H)-naphthalene]-5′-carboxylic acid	Terpenoids	C_20_H_30_O_4_	333.2288/235.8418/137.8908	-
23	9.091	356	Rutamarin	Coumarins	C_21_H_24_O_5_	355.1585/119.9463	[37]
24	9.681	598	12b-O-[deca-2Z,4E-dienoyl]-13a-isobutyl-5-ene-7-oxo-4b-phorbol	Aromatic derivatives	C_34_H_46_O_9_	621.3096/533.2560/434.2414	-
25	9.772	390	2-acetoxy-4-pentadecylbenzoic acid	Aliphatic derivatives	C_24_H_38_O_4_	413.2644/301.1388	-

Peak No. is the same in Figure 3; RT indicates retention time.

#### 2.3.2. Volatile Compound Analysis

**Figure 6:**
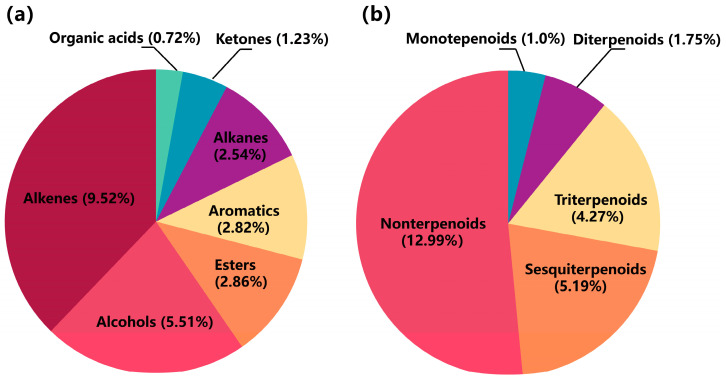
Relative content of identified volatile compounds. (**a**) Relative content of compounds classified by functional groups. (**b**) Relative content of different terpenoid types.

For the comprehensive identification of volatile components, GC-MS was performed using an optimized temperature program and split ratio. The total ion chromatogram result is shown in Figure 5. Based on the retention index and mass spectra information, compounds were identified by comparing them to the NIST 2020 library database. As a result, 39 volatile compounds were assigned to terpenoids, phytosterols, aromatic derivatives, and aliphatic derivatives, accounting for 25.00% of the total content (Figure 6a). Moreover, terpenoids had the highest content, comprising 12.21%, including monoterpeneoids (1.0%), sesquiterpenoids (5.19%), diterpenoids (1.75%), and triterpenoids (4.27%). Among them, the compounds with the highest contents were estragole, γ-muurolene, phytol, and squalene (Figure 6b). Aromatic and aliphatic derivatives, such as 2,4-di-tert-butylphenol and 2-hexadecen-1-ol, 3,7,11,15-tetramethyl-acetate (a diterpenoid derivative), accounted for 1.56% and 1.50% of the total content, respectively. The phytosterols includes campesterol and stigmasterol, with 1.00% and 2.85% of the total content, respectively (Table 4).

## 3. Discussion

*H. rhomboidea*, a traditional herbal medicine used to treat hepatitis, has been investigated for its chemical constituents, antimicrobial activity, and cytotoxicity activity against the murine tumor cell line in few studies [14,38]. We evaluated the cytotoxic activity of *H*. *rhomboidea* ethanol extract on five human cancer cell lines for the first time. The results indicated that the 95% ethanol extract of *H*. *rhomboidea* (HR-95) exhibits pronounced inhibitory activity, while the 50% extract (HR-50) shows no significant effect. However, HR-50 significantly inhibits NO production in murine monocyte–macrophages, indicating its excellent anti-inflammatory effects. At a concentration of 50 μg/mL, HR-50 achieved an inhibition rate of 97.23% on NO production. Nevertheless, it also exhibited cytotoxicity (Table S1). In addition, both of the extracts (HR-95 and HR-50) showed no cytotoxic against non-cancer cells (normal human lung epithelial cells), which demonstrated that the *H*. *rhomboidea* extracts have selectivity against human cancer cells. This finding indicates that the potential cytotoxicity of the ethanol extract from *H*. *rhomboidea* are relative to the polarity of its metabolites, and the big polar compounds from the 50% ethanol extract may associate with their anti-inflammatory activity. These results support the medicinal value of *H. rhomboidea*.

The MTS assay revealed that the 95% ethanol extract of *H. rhomboidea* exhibited lower cytotoxic activity against the five human cancer cell lines compared with the positive controls (cisplatin and Taxol), yet it still demonstrated a moderate level (the extract showed over half inhibitory rate at 100 µg/mL) of an inhibitory effect, with IC_50_ values ranging from 15.8 μg/mL to 40.0 μg/mL. This may be attributed to the low concentration of the bioactive metabolites and the interaction of multiple compounds. The separation and purification of bioactive compounds can enhance their cytotoxicity against cancer cells. Consequently, the chemical composition of HR-95 was investigated using LC-MS and GC-MS.

In this study, we employed LC-MS and GC-MS methods to analyze and characterize the chemical composition of HR-95, resulting in the identification of various potential biologically active compounds. Among them, flavonoids were the main components in *H. rhomboidea* crude extract. Previous studies have reported that flavonoids possess extensively cytotoxicity against cancer cells [39,40]. Studies have found that 3′,4′,5′-trimethoxyflavonol induces apoptosis in TRAMP C2 prostate cancer cells [41]. Quercetin not only triggers apoptosis in human colon cancer cells by inhibiting the nuclear factor-kappa B pathway but also suppresses proliferation and invasion in breast cancer cells through the up-regulation of miR-146a. It further induces autophagy-associated cell death in HL-60 cells via the CaMKKβ/AMPK/mTOR signaling pathway and exhibits other anti-tumor effects as well [42,43,44,45]. Kaempferol induces the cell cycle at the G2/M phase in MDA-MB-453 breast cancer cells and reverses the EMT process in gastric, ovarian, and breast cancer cells by modulating key markers such as E-cadherin, Smad2/4, TGF-β, N-cadherin, vimentin, and Snai1 [46,47]. Astragalin has been shown to effectively inhibit the proliferation of leukemia (HL-60), hepatocellular (HepG2), skin (HaCaT), and lung (A549) cancer cells [48]. Meanwhile, genkwanin demonstrates strong efficacy in inhibiting the proliferation and metastasis of A549 and H69AR cancer cells and shows potential in treating colon cancer and B16F10 melanoma [49]. The effective inhibition of human tumor cells by the *H. rhomboidea* extract may be attributed to these compounds.

Furthermore, rosmarinic acid, corosolic acid, rutin, spiraeoside, rutamarin, and some other compounds also demonstrated definite inhibitory activity on tumor cells [50,51,52,53,54]. Terpenoids were the main components of volatile compounds, and squalene (triterpenoids) exhibited notable cytotoxic effect on cancer cells [55]. Additionally, phytosterols, i.e., campesterol and stigmasterol, also possessed cytotoxic activity against human cancer cells [56,57]. These compounds may inhibit the synthesis of proteins and the replication of DNA in cancer cells to promote the cytotoxic activity of HR-95 against human cancer cell lines [58].

Previous studies have found that the main components of *H*. *rhomboidea* are phenolic acids and glycosides, which have inhibitory effects on plant pathogens such as *Fusarium graminearum* and *Exserohilum turcicum* [38]. The ethanol extract can improve the levels of serum TNF-α and IL-2 in mice, boost SOD activity, and decrease MDA content; thus, it can against the growth of digestive tumor cells with an inhibition activity of 68.84% [14]. However, there are few reports on the chemical composition of *H*. *rhomboidea* and their cytotoxicity against human cancer cells. In this study, we observed satisfactory activity of the ethanol extract of *H*. *rhomboidea* on human cancer cells for the first time. Subsequently, using metabolomics approaches with UPLC-QTOF-MS and GC-MS, we analyzed the chemical composition of the extract and identified potential active compounds associated with cytotoxicity, providing new insights into the isolation and purification of bioactive compounds. Moreover, merging bioactivity with metabolomic techniques can provide an effective strategy to discover bioactive compounds from medicinal plants [18], thereby addressing the limitations of traditional methods in isolating bioactive compounds.

## 4. Materials and Methods

### 4.1. Plant Materials

The *H. rhomboidea* plant was collected from Ledong county, Hainan province, China. Voucher specimens were verified by Dr. Haijun Yang (South China Agricultural University) and stored at −40 °C in the laboratory of chemical ecology until extraction. 

### 4.2. Perparation of Crude Extract

The aerial part of *H. rhomboidea* was naturally air-dried and ground into a powder. The dried powder was weighed to 200 g accurately and evenly divided into 2 portions (100 g each). Subsequently, samples are extracted with 50% ethanol and 95% ethanol for 48 h and then sonicated at 20 °C for 30 min. Each sample underwent two extractions using the same method. The filtrate was concentrated with a vacuum rotary evaporator to produce dry residues. The 50% ethanol crude extract was 328 mg, and the 95% ethanol crude extract was 169 mg. The extracts were stored at −20 °C until use.

### 4.3. Cytotoxic Activity Assay

Human cancer cell lines HL-60 (leukemia), MDA-MB-231 (breast cancer), A-549 (lung cancer), and SW480 (colon cancer) were obtained from ATCC (Manassas, VA, USA), and SMMC-7721 (hepatoma) and BEAS-2B (normal human lung epithelial cells) were sourced from BeNa Culture Collection (BNCC, Beijing, China). The cells were cultured in either RMPI-1640 or DMEM medium (Biological Industries, Kibbutz Beit-Haemek, Israel) supplemented with 10% fetal bovine serum for a duration of 12 to 24 h [59]. The cytotoxic activity of the cancer cells was assessed using the MTS assay [60]. In short, the cells were diluted with medium to obtain a single-cell suspension and then inoculated to a 96-well plate. The crude extract (100 µg/mL, prepared in DMSO), the solvent DMSO (blank controls), 12.00 µg/mL (40 µM) cisplatin, and 4.27 µg/mL (5 µM) Taxol (positive controls) were added to 100 µL of the medium, resulting in a final volume of 200 μL and an initial concentration of 1 × 10^5^ cells/mL. After incubating for 48 h at 37 °C, 100 μL of the culture medium was discarded and supplemented with 20 μL of the MTS solution. Each treatment was performed in triplicate. The treatments showing over 50% growth inhibition were further evaluated at different concentrations of the crude extracts, cisplatin, and Taxol. Specifically, the crude extract concentrations were 100 µg/mL, 20 µg/mL, 4 µg/mL, and 0.8 µg/mL. Furthermore, the cisplatin concentrations were 12.00 µg/mL (40 µM), 2.40 µg/mL (8 µM), 0.48 µg/mL (1.6 µM), 0.096 µg/mL (0.32 µM), and 0.019 µg/mL (0.064 µM), while the Taxol concentrations were 4.27 µg/mL (5 µM), 0.85 µg/mL (1 µM), 0.17 µg/mL (0.2 µM), 0.034 µg/mL (0.04 µM), and 0.0068 µg/mL (0.008 µM). Each treatment was tested in triplicate. After 48 h of cell incubation by the aforementioned protocol, the treatments were further incubated for an additional 4 h to ensure sufficient reaction time. Following incubation, cell viability was assessed by measuring the absorbance of the treatments at a wavelength of 492 nm using a Multiskan FC Microplate Photometer (Thermo Fisher Scientific, Waltham, MA, USA). The IC_50_ value of each treatment was determined with Reed and Muench’s method, followed by analysis and drawing using Origin 2022.

### 4.4. UPLC-QTOF-MS Analysis of Non-Volatile Compounds

The 95% ethanol crude extract of *H. rhomboidea* was dissolved in methanol to a final concentration of 1 mg/mL and filtered through a 0.22 μm PTFE filter for analysis [61]. The prepared samples were analyzed using an ACQUITY UPLC system connected to a Xevo G2-XS QTOF spectrometer (Waters Milford, Milford, MA, USA). Separation was achieved on an ACQUITY UPLC^®^ BEH C18 column (2.1 mm × 50 mm, 1.7 µm; Waters) maintained at 40 °C, with an injection amount of 2 μL. The elution utilized a two-solvent system as follows: solvent A was water with 0.1% formic acid, and solvent B was acetonitrile with 0.1% formic acid. The gradient program was as follows: 0–1.0 min, 10% B; 1.0–2.0 min, 10~30% B; 2.0–8.0 min, 30–98% B; 8.0–10.0 min, 98% B; 10.0–10.2 min, 98~10% B; 10.2–13.0 min, and 10% B. The flow rate was set to 0.3 mL/min, and the autosampler temperature was maintained at 16 °C. High-resolution mass spectrometry was detected in both negative and positive ion sensitivity mode with an electrospray ionization source. The essential parameters of the Xevo G2-XS QTOF-MS included a capillary voltage of +2.50 kV and −1.5 kV, source temperature of 120 °C, desolvation temperature of 350 °C, cone gas flow rate of 50 L/h, desolvation gas flow rate of 800 L/h, scan time of 0.5 s, mass range of 100–1000 *m*/*z*, low energy of 5 V, and collision energy of 20~50 V. All compounds were detected using MS^E^ mode to obtain fragmentation data in a single analysis. 

### 4.5. GC-MS Analysis of Volatile Compounds

The 95% ethanol crude extract of *H. rhomboidea* was dissolved with methanol (with 5 mg/L ethyl caprate) to a concentration of 1 mg/L. Subsequently, the extracts were sonicated for 30 min in a 15 °C water bath, followed by drying over anhydrous sodium sulfate and stored at −4 °C for 12 h. Then, 1 mL of extraction solution was aspirated and filtered through a 0.22 μm PTFE into a 2 mL sample vial for GC-MS analysis. 

The GC-MS analysis was conducted using a SHIMADZU TQ8040 GC-MS instrument, which featured a triple quadrupole configuration and a DB-5MS column (30 m × 0.25 mm × 0.25 μm, Agilent Technologies, Santa Clara, CA, USA). The GC parameters were as follows: helium was used as the carrier gas at a flow rate of 1 mL/min, the injection temperature was set to 230 °C, the detector temperature was 250 °C, the sample volume was 1 µL, and the split ratio was 3: 1. The oven temperature was increased using the following procedure: starting at 60 °C for 2min, ramped at 6 °C/min to 250 °C and kept for 15 min, then maintained 250 °C for 10 min. The MS utilized an EI ionization source operating at 70 eV, with a scan range of 50–500 *m*/*z*. The ion source temperature was set at 200 °C, and the GC-MS interface temperature was maintained at 250 °C. The raw compound data files were converted from QGD format to CDF format using SHIMADZU GSMSsolution (Ver. 4.52) for subsequent analysis.

### 4.6. Data Analysis

The raw data acquired from UPLC-QTOF-MS and GC-MS analyses were initially converted to ABF format files by ABF converter (Ver. 1.3). Subsequently, compound relative content, mass spectra information, relative retention index (RI), and relative retention time (RT) were obtained using MSDAIL (Ver. 4.9) for compound identification. The MSDAIL parameters were set to default. The non-volatile compounds of UPLC-MS were identified using the Massbank library, MSDAL standard compound library, METVARE self-built library, and published mass spectra. Meanwhile, the volatile compounds of GS-MS were identified using the NIST 2020 mass spectral library and published mass spectra, and the compounds with matching ≥ 800 were retained.

## 5. Conclusions

In this study, we first discovered that the 95% ethanol extract of *H. rhomboidea* exhibited moderate cytotoxic activities against five human cancer cell lines (HL-60, A549, SMMC7721, MDA-MB-231, and SW480), with IC_50_ values ranging from 15.8 to 40.0 μg/mL. Based on metabolomic analysis using UPLC-QTOF-MS and GC-MS methods, a total of 25 non-volatile and 39 volatile compounds from the active crude extract were identified, and several compounds had the potential cytotoxicity need further isolation and verification. These findings highlight the cytotoxic potential of *H. rhomboidea* and provide valuable insights for its further exploration and application in cancer research and therapeutic development.

## Figures and Tables

**Table 1:** Inhibitory rate (%) of two *H. rhomboidea* ethanol extracts on five types of human cancer cells.

Groups	HL-60	A549	SMMC-7721	MDA-MB-231	SW480	BEAS-2B
HR-95	96.10 ± 0.13	78.24 ± 0.90	79.43 ± 1.35	90.54 ± 1.01	85.64 ± 0.26	14.20 ± 1.43
HR-50	−41.52 ± 1.03	3.64 ± 1.64	20.40 ± 0.57	12.35 ± 2.92	16.24 ± 1.11	1.27 ± 0.68

HR-95 means the 95% ethanol extract of *H. rhomboidea*; HR-50 means the 50% ethanol extract of *H. rhomboidea*. The extract concentration was 100 μg/mL. BEAS-2B is human normal lung epithelial cells (non-cancer cell line), and the values are expressed as mean ± SD of cell inhibition rate (*n* = 3).

**Table 2:** The IC_50_ value (μg/mL) of *H. rhomboidea* crude extract on five human cancer cell lines.

Groups	HL-60	A549	SMMC-7721	MDA-MB-231	SW480	BEAS-2B
HR-95	36.44 ± 1.82	40.02 ± 0.58	26.20 ± 0.77	22.95 ± 1.59	15.81 ± 0.63	>100
Cisplatin	4.25 ± 0.22	5.40 ± 0.12	5.05 ± 0.20	5.07 ± 0.39	7.52 ± 0.38	23.44 ± 1.14
Taxol	<0.007	<0.007	0.466 ± 0.25	<0.007	<0.007	2.44 ± 0.42

HR-95: 95% ethanol extract of *H. rhomboidea*. Cisplatin and Taxol were used as positive controls. The values are expressed as mean ± SD of IC_50_ (*n* = 3).

**Table 4:** Essential compounds in the 95% ethanol extracts of *Hyptis rhomboidea* extracted using GC-MS.

Peak No.	RT (min)	Compound	Molecular Formula	Library No.	Match	Retention Index		Relative Content (%)
						Average RI	Lib.RI	
1	7.495	m-Cymene	C_10_H_14_	535-77-3	801	1026	1023	0.16%
2	8.155	γ-Terpinene	C_10_H_16_	99-85-4	838	1060	1060	0.13%
3	8.940	Linalool	C_10_H_18_O	78-70-6	848	1100	1099	0.28%
4	10.455	endo-Borneol	C_10_H_18_O	507-70-0	902	1178	1167	0.21%
5	10.915	Estragole	C_10_H_12_O	140-67-0	890	1201	1196	0.22%
6	14.175	α-Ylangene	C_15_H_24_	14912-44-8	880	1378	1372	0.14%
7	14.290	(-)-alpha-Copaene	C_15_H_24_	3856-25-5	895	1384	1376	0.62%
8	15.090	Caryophyllene	C_15_H_24_	87-44-5	845	1404	1419	0.18%
9	15.710	Humulene	C_15_H_24_	6753-98-6	839	1467	1454	0.21%
10	15.985	γ-Muurolene	C_15_H_24_	30021-74-0	927	1483	1477	0.98%
11	16.060	α-Muurolene	C_15_H_24_	31983-22-9	919	1488	1499	0.14%
12	16.275	β-Eudesmene	C_15_H_24_	17066-67-0	836	1500	1486	0.24%
13	16.405	2,4-Di-tert-butylphenol	C_14_H_22_O	96-76-4	870	1509	1519	1.56%
14	16.630	γ-Cadinene	C_15_H_24_	39029-41-9	939	1523	1513	0.79%
15	16.690	δ-Cadinene	C_15_H_24_	483-76-1	855	1526	1524	0.49%
16	16.760	Calamenene	C_15_H_22_	483-77-2	870	1531	1523	0.38%
17	17.010	α-Cadinene	C_15_H_24_	24406-05-1	870	1547	1538	0.20%
18	17.820	Caryophyllene oxide	C_15_H_24_O	1139-30-6	800	1597	1581	0.32%
19	18.435	Muurola-4,10(14)-dien-1β-ol	C_15_H_24_O	257293-90-6	844	1638	1635	0.44%
20	18.685	10-epi-α-Cadinol	C_15_H_26_O	1474790	870	1654	1580	0.06%
21	21.305	Neophytadiene	C_20_H_38_	504-96-1	920	1836	1837	0.64%
22	21.385	Hexahydrofarnesyl acetone	C_18_H_36_O	502-69-2	904	1842	1844	1.23%
23	21.675	Diisobutyl phthalate	C_16_H_22_O_4_	84-69-5	877	1863	1870	0.15%
24	21.895	Neophytadiene	C_20_H_38_	504-96-1	910	1879	1774	0.26%
25	22.350	Farnesyl palmitate	C_31_H_56_O_2_	157501-12-7	810	1913	3225	0.18%
26	22.940	Dibutyl phthalate	C_16_H_22_O_4_	84-74-2	825	1959	1965	0.09%
27	22.980	n-Hexadecanoic acid	C_16_H_32_O_2_	57-10-3	851	1962	1968	0.72%
28	23.360	Hexadecanoic acid, ethyl ester	C_18_H_36_O_2_	628-97-7	903	1991	1993	0.21%
29	24.850	Phytol	C_20_H_40_O	150-86-7	892	2111	2114	0.67%
30	26.035	2-Hexadecen-1-ol, 3,7,11,15- Tetramethyl-, acetate	C_22_H_42_O_2_	76337-16-1	911	2212	2232	1.50%
31	26.395	Tributyl acetylcitrate	C_20_H_34_O_8_	77-90-7	801	2243	2250	0.14%
32	27.625	4,8,12,16-Tetramethylheptadecan -4-olide	C_21_H_40_O_2_	96168-15-9	804	2354	2364	0.28%
33	28.645	Unknown	-	-	-	-	-	19.86%
34	29.530	Unknown	-	-	-	-	-	14.39%
35	31.370	Heptacosane	C_27_H_56_	593-49-7	857	2699	2700	1.14%
36	32.960	Squalene	C_30_H_50_	111-02-4	925	2816	2832	4.27%
37	34.290	Nonacosane	C_29_H_60_	630-03-5	830	2907	2900	0.94%
38	38.550	Dotriacontane	C_32_H_66_	544-85-4	820	3197	3200	0.47%
39	39.385	Vitamin E	C_29_H_50_O_2_	59-02-9	940	3254	3149	0.73%
40	42.480	Campesterol	C_28_H_48_O	474-62-4	800	3465	3131	1.00%
41	43.320	Stigmasterol	C_29_H_48_O	83-48-7	840	3522	3170	2.85%

RT: retention time. RI: retention index. Library No. includes the CAS ID and NIST ID of compounds. Relative content is calculated by peak area/total peak area of compounds.

## Data Availability

Data are contained within the article and Supplementary Materials.

## Supplementary Materials

The following supporting information can be downloaded at https://www.mdpi.com/article/10.3390/molecules29174216/s1: Table S1. The inhibitory rate of nitric oxide (NO) generation for two *H. rhomboidea* ethanol extracts and the positive control.
