# Supplementary material for: The Cytotoxic Activity and Metabolic Profiling of Hyptis rhomboidea Mart. et Gal
Source: Molecules. 2024 Sep 5;29(17):4216. doi: 10.3390/molecules29174216 (PMC11396782; doi:10.3390/molecules29174216)
Supplement: Supplementary file 1 [file molecules-29-04216-s001.zip › molecules-3178456-supplementary.pdf]

Supplementary Information

Table S1 The inhibitory rate of nitric oxide (NO) generation for two *H. rhomboidea* ethanol extracts and positive control

Groups	Concentration	Inhibitory rate of NO generation (%)
L-NMMA	50μM	56.98±0.90
HR-95	50μg/ml	16.24±1.70
HR-50	50μg/ml	97.23±0.63（cytotoxicity）
HR-50	12.5μg/ml	51.74±0.62

HR-95 means 95% ethanol extract of *H. rhomboidea*, HR-50 means 50% ethanol extract of *H. rhomboidea*. L-NMMA means positive control, named tilarginine acetate.

Screening assay of nitric oxide (NO) production inhibitors

Chemicals and materials

A murine monocyte-macrophage RAW264.7 cell line was purchased from the Cell Bank of the Chinese Academy of Sciences in Shanghai. Dulbecco's Modified Eagle's Medium (DMEM) and Fetal Bovine Serum (FBS) was obtained from Biological Industries (Israel). Griess Reagent, LPS and L-NMMA (positive control drug) were acquired from Sigma–Aldrich (St., Louis, MO, USA).

Measurement method of NO

Induce the production of nitric oxide (NO) synthase in murine monocyte-macrophage RAW264.7 cells by treating them with lipopolysaccharide (LPS), and simultaneously add the test compound for treatment. Measure the absorbance of nitrite (NO^{2-}) in the culture medium at a wavelength of 570nm using the Griess assay to detect the generation of nitric oxide.

Shortly, RAW 264.7 cells were seeded in 96-well plate and incubated with LPS (1μg/mL) at 37 °C, while simultaneously adding the test compound at a final concentration of 50 μg/mL for treatment. Control groups were set up, including a group without the test compound and a positive drug group with L-NMMA. After overnight incubation, the culture medium was collected to detect nitric oxide (NO) production. Each cell culture supernatant (100 μL) was mixed with the same volume of Griess reagent for 10 min at room temperature. The absorbance was measured at 570 nm and nitrite concentrations in the supernatant were determined by comparison with a sodium nitrite standard curve. MTS was added to the remaining culture medium to assess cell viability and to exclude any potential toxic effects of the compound on the cells.

Inhibitory rate of NO Production = ($OD_{570\ nm}$ of Non-treatment Group - $OD_{570\ nm}$ of Sample Group) / $OD_{570\ nm}$ of Non-treatment Group × 100%
